# Evaluation of mutagenesis, necrosis and apoptosis induced by omeprazole in stomach cells of patients with gastritis

**DOI:** 10.1186/s12935-022-02563-5

**Published:** 2022-04-18

**Authors:** Ana Maria Oliveira Ferreira da Mata, Marcia Fernanda Correia Jardim Paz, Ag-Anne Pereira Melo de Menezes, Antonielly Campinho dos Reis, Bruna da Silva Souza, Carlos Dimas de Carvalho Sousa, Sônia Alves Machado, Thiago Soares Gondim Medeiros, Chandan Sarkar, Muhammad Torequl Islam, Javad Sharifi-Rad, Sevgi Durna Daştan, Mohammed M. Alshehri, João Marcelo de Castro e Sousa, Ana Amélia de Carvalho Melo Cavalcante

**Affiliations:** 1 Postgraduate Program in Biotechnology (RENORBIO), Federal University of Piauí, Teresina, PI 64.049-550 Brazil; 2 Federal University of Delta Do Parnaíba, Parnaiba, PI 64202-020 Brazil; 3 Getúlio Vargas Hospital, Teresina, PI 64001-020 Brazil; 4 Department of Pharmacy, Bangabandhu Sheikh Mujibur Rahman Science and Technology University Bangladesh, Gopalganj, 8100 Bangladesh; 5 Phytochemistry Research Center, Shahid Beheshti University of Medical Sciences, Tehran, Iran; 6 Facultad de Medicina, Universidad del Azuay, Cuenca, Ecuador; 7 Department of Biology, Faculty of Science, Sivas Cumhuriyet University, 58140 Sivas, Turkey; 8 Beekeeping Development Application and Research Center, Sivas Cumhuriyet University, 58140 Sivas, Turkey; 9 Pharmaceutical Care Department, Ministry of National Guard-Health Affairs, Riyadh, Saudi Arabia

**Keywords:** Citogenetic biomarkers, Oxidative stress, Apoptosis, Genetic instability

## Abstract

**Background:**

Gastritis is a superficial and prevalent inflammatory lesion that is considered a public health concern once can cause gastric ulcers and gastric cancer, especially when associated with *Helicobacter pylori* infection. Proton pump inhibitors, such as omeprazole, are the most widely used drugs to treat this illness. The aim of the study was evaluate cytogenetic effects of omeprazole in stomach epithelial cells of patients with gastritis in presence and absence of *H. pylori*, through cytogenetic biomarkers and catalse and superoxide dismutase analysis.

**Methods:**

The study included 152 patients from the Gastroenterology Outpatient Clinic of Hospital Getúlio Vargas, Teresina—Brazil, that reported continuous and prolonged omeprazole use in doses of 20, 30 and 40 mg/kg. The participants were divided into groups: (1) patients without gastritis (n = 32); (2) patients without gastritis but with OME use (n = 24); (3) patients with gastritis (n = 26); (4) patients with gastritis undergoing OME therapy (n = 26); (5) patients with gastritis and *H. pylori* (n = 22) and (6) patients with gastritis and *H. pylori* on OME therapy (n = 22).

**Results:**

OME induced cytogenetic imbalance in the stomach epithelium through the formation of micronuclei (group 6 > 1, 2, 3, 4, 5; group 5 > 1, 2, 3; group 4 > 1, 2, 3); bridges (groups 4 and 6 > 1, 2, 3, 5 and group 2 > 3, 5); buds (groups 2,4,6 > , 1, 3, 5); binucleated cells (group 6 > 1, 2, 3, 4, 5; group 4 > 1, 2, 3); (groups 2 and 3 > 1); picnoses (group 6 > 1, 2, 3, 4, 5), groups 2 and 5 > 1, 3; group 4 > 1, 2, 3, 5); cariorrexis (groups 6 and 4 > 1, 2, 3, 5; groups 2, 3, 5 > 1) and karyolysis (groups 2, 4, and 6 > 1, 3, 5; groups 3 and 5 > 1). The OME cytogenetic instability was associated with *H. pylori* infection, indicating clastogenic/aneugenic effects, chromosomes alterations, gene expression changes, cytotoxicity and apoptosis.

**Conclusions:**

The cytogenetic changescan be attributed to several mechanisms that are still unclear, including oxidative damage, as observed by increased catalase and superoxide dismutase expresion. Positive correlations between antioxidant enzymes were found with micronuclei formation, and were negative for picnoses. Thus, the continuous and prolonged omeprazole use induces genetic instability, which can be monitored through cytogenetic analyzes, as precursor for gastric cancer.

## Introduction

Gastric lesions, such as gastritis, can damage the gastric wall and/or duodenal epithelium, producing ulcers and even cancer [27, 43, 49]. Other pathological gastric changes may occur due to *Helicobacter pylori* (*H. pylori*) co-infection, which increase hydrochloric acid and pepsin secretion by the gastric mucosa [27, 43]. Proton Pump Inhibitors (PPIs) such as Omeprazole (OME), lansoprasol, pantoprazole, esomeprazole, among others are used to suppress gastric acidity [55, 64, 88], and have been used in long-term therapies [16, 17, 46, 70].

PPIs cause several adverse effects, and there are reports that its prolonged use promotes cardiovascular and renal alterations, anemia, thrombocytopenia, gastric polyps and carcinoma [31, 40, 94, 107]. In addition, PPIs can induce DNA fragmentation [29], causing chromosome rearrangements [91], as well as chromosomes breaks, which increase micronuclei formation (MN) [8, 84]. Genotoxic drugs lead to tMN formation [1, 36] and other types of nuclear alterations such as apoptosis, cytoplasmic bridges and nuclear shoots [33, 34]. Therefore, cytogenetic evaluations are important for several human diseases, as well as for therapeutic monitoring of genotoxicity [11, 48, 87].

Drug metabolic products can induce genetic mutations, breaks and/or chromosome rearrangements. There are reports of OME metabolites cytotoxicity, hepatoxicity and carcinogenicity [91]. Cytochrome P450 enzymes (CYPs) act on PPIs metabolism [26], which are considered monoxygenases responsible for the metabolism of several drugs through expodification, hydrolyzation, desulfurization, dealkylation, oxidation or sulfoxidation reactions [6]. OME is biotransformed in the liver by enzymatic activity of CYP2C19 and CYP3A4, producing the 5-hydroxy (5-OH) omeprazole and omeprazole Sulfona metabolites [30, 71].

In non-clinical studies, OME toxicogenic effects were identified in plant cells (*Allium* cepa) [13], as well as *Saccharomyces cerevisiae* and murine Sarcoma 180 cells [75]. Thus, based in previous investigations, the present study aimed to evaluate cytogenetic damages in patients whith gastritis undergoing OME therapy, with and without *H. pylori* co-infection, through micronucleus test and expression of catalase and superoxide dismutase.

## Materials and methods

### Ethical aspects

The present study was a controlled cross-sectional research, approved by the Research Ethics Committees (CEP) of UNINOVAFAPI (no. 1.521.307), Federal University of Piauí—UFPI (no. 1.607.441) and Ethics Committee of Hospital Getúlio Vargas (No. 1,569,041). All participants agreed to participate voluntarily and signed the Free and Informed Consent Form (FICF), in accordance with resolution 466/12 of the National Health Council.

### Study location and sample

One hundred and fifty-two patients from the Gastroenterology Outpatient Clinic of Hospital Getúlio Vargas, Teresina—PI (2017–2019) were enrolled in this study. The participants had been undergone to upper digestive endoscopy and reported prolonged OME use (or not) in doses of 20 and 40 mg/kg. Medical reports about the presence or absence of gastric diseases, including gastritis and *H. pylori* infection, were examined after endoscopy and urea test. Participants were grouped according to the criteria: WG—patients without gastritis (n = 32); WG + OME—patients without gastritis with OME use (n = 24); G—patients with gastritis (n = 26); G + OME—patients with gastritis and OME use (n = 26); G + HP—patients with gastritis and *H. pylori* (n = 22) and G + HP + OME—patients with gastritis and *H. pylori*, using OME (n = 22).

### Inclusion and exclusion criteria

The study included patients: (1) with or without gastritis; over 18 years old and legally responsible; (2) that signed the informed consent form; (3) that have prolonged OME useor not, by medical recommendation or self-medication, or reported no OME use;. The following participants were excluded: (1) over 70-year-old; (2) current illnesses that required surgical treatment, chemotherapy or radiotherapy.

### Data collection

After Informed Consent Form signed, questionnaires were applied according to Carrano and Natarajam [21] based on the protocol published by the International Commission for Environmental Protection against Mutagens and Carcinogens (ICPEMC), with adaptations for nutritional aspects, socio-cultural and health and lifestyle information.

Exfoliated cells from the oral epithelium of patients undergoing endoscopy were obtained by scraping the inside of the cheek with a cytobrush. Cells of gastric epithelium (region of the body and antrum of the stomach) were collected at the time of endoscopy. All samples were placed in tubes with sodium phosphate buffer (PBS) (50 mM, pH 7.4), properly identified, and transported in dry ice to the Laboratory of Toxicological Genetics of the Federal University of Piauí, for immediately processing and tests. Peripheral blood samples were collected with heparin, and transported similarly to the other samples. The urea test was performed by the hospital’s medical team and the results were released together with the medical report.

### Micronucleus test on exfoliated cells of the stomach epithelium

The Micronucleus Test was performed according to Thomas et al. [95], with some adaptations. Samples of the stomach epithelium (antrum and body) were collected during endoscopy. The material collected was placed in identified and previously prepared microtubes, containing 5 mL of saline solution (0.9% NaCl). Immediately after, the samples were sent to the Laboratory of Toxicological Genetics at the Federal University of Piauí in for analysis. To avoid external contamination, cell samples were washed three times before smear preparation. The washing process was carried out in 5 mL saline solution (0.9% NaCl), with centrifugations for 10 min at 1500 rpm, followed by removal of the supernatant and replacement of the solution always in the final volume of 5 mL. Two slides were prepared for each patient. After fixation, with methanol/acetic acid (3:1), the slides were stained with 2% Giemsa. Then, the slides were washed twice in distilled water for 3 min and, finally, dried at room temperature. The incidence of micronuclei, nuclear buds, binucleated cells and nuclear abnormalities that represent cell death, carioretic, pycnotic and karyolitic cells, were observed in 2000 cells per patient with the use of optical microscopy, in the 1000 × amplification.

### Profile of patients’ enzymatic antioxidant defenses

From the peripheral blood samples collected, 10% erythrocyte homogenates were prepared (50 mM sodium phosphate buffer pH 7.4), which were centrifuged (800*g*, 20 min) and the supernatants used for catalase (CAT) activity assay. The reaction medium was prepared with H_2_O_2_ (18 mL) plus 1 M Tris HCl Buffer, 5 nM EDTA pH 8.0 (1.0 mL) and H2O (0.8 mL). Then, 980 μL of the reaction medium and 20 μL of the 10% erythrocyte homogenate was placed in the quartz cuvette. Finally, the reading was performed in a spectrophotometer for 6 min at 230 nm. The blank was made by reading the relative absorbance at 230 nm with only 1 mL of the reaction medium [23]. The protein concentration was determined [58]. The results were expressed in mmol/min/mg of protein. The homogenates of the 10% erythrocytes (50 mM sodium phosphate buffer, pH 7.4) were also centrifuged (800*g*, 20 min) and the supernatants used for superoxide dismutase (SOD) activity assessment. SOD activity was tested using the reduction rate of cytochrome C by superoxide radicals, using the xanthine-xanthine oxidase system as a source of superoxide anion (O_2_^−^) [5]. The results were expressed in U/mg of protein. One unit (U) of SOD activity corresponds to a 50% inhibition of the reaction of O_2_^−^ with cytochrome C. For protein concentration, the method of Lowry et al. [58] was used.

### Urease test

The evaluation of H*. pylori* presence was performed by the urea test, according to Uotani and Graham [103]. Samples of gastric epithelial mucosa (antrum and body) were collected by biopsy during endoscopy. The urease test was performed and the result was obtained together with the endoscopy report.

### Statistical analysis

The results of the analyzed biomarkers were presented as mean ± the standard deviation from mean. The data obtained were evaluated using Analysis of Variance (ANOVA) followed by the Bonferroni test as a post hoc test. The data were analyzed using the GraphPad Prism 6.0 software (San Diego, CA, USA), the experimental groups were compared with the control group and with each other. Pearson's correlations were performed using the IBM SPSS Statistics 23 statistical program. P < 0.05 was defined as statistically significant.

## Results

After applying the questionnaire depicting nutritional aspects, socio-cultural, health and lifestyle information for each patient, the investigated population was characterized (Table 1). The patients were aged 36–53 years old, mostly female, brown colored people, married and lower education level. Most participants were not exposed to potentially mutagenic chemicals, such as pesticides, cleaning materials, dyes and solvents. However, it was observed that 64% of patients with G + OME reported exposure to cleaning products and 44% to pesticides. Patients reported no regular physical exercises, as well low alcohol consumption, smoking, meat and vegetables consumption. In addition, more than 50% of patients with gastritis, including those with *H. pylori* coinfection, reported family history of cancer and absence of other hereditary diseases.

**Table 1:** Sociocultural and health characteristics of patients with gastritis and on omeprazole (OME) therapy at Getúlio Vargas Hospital

Parameters	WG (n = 26)	G (n = 23)	G + HP (n = 16)	WG + OME (n = 22)	G + OME (n = 25)	G + HP + OME (n = 25)
Gender (% valid)						
Male	26.9	34.8	50.0	40.9	44.0	12.0
Female	73.1	65.2	50.0	59.1	56.0	88.0
Ethnic groups (% valid)						
White	11.5	17.4	12.5	27.3	20.0	48.0
Pardo^a^	57.70	56.5	62.5	63.6	64.0	36.0
Black	30.8	26.1	25.0	4.5	16.0	16.0
Age(MD ± DV)	48.46 ± 13.98	53.57 ± 17.08	39.81 ± 17.17	36.36 ± 11.99	51.48 ± 11.99	51.40 ± 13.21
Weight (kg) (MD ± DV)	60.31 ± 9.39	62.78 ± 13.54	67.20 ± 14.65	62.40 ± 8.42	66.73 ± 11.76	68.44 ± 8.99
Marital status (% valid)						
Married	42.3	30.4	37.5	45.5	60.0	60.0
Divorced	11.5	26.1	25.0	4.5	8.0	28.0
Single	30.8	26.1	37.5	50.0	20.0	–
Widowed	15.4	17.4	–	–	12.0	12.0
Education level (% valid)						
Without education level complete	19.2	13.0	12.5	–	24.0	12.0
Elementery	11.5	–	31.3	18.2	24.0	12.0
Elementery(incomplete)	19.2	52.2	6.3	31.8	24.0	12.0
High school	19.2	17.4	50.0	36.4	12.0	52.0
High school (incomplete)	13.8	8.7	–	–	16.0	12.0
Bachelor	–	8.7	–	–	–	–
Bachelor (incomplete)	–	–	–	9.1	–	–
Chemical exposure (% valid)						
Cleaning product	23.1	30.4	60.0	50.0	64.0	60.0
Agrochemicals	15.4	13.0	12.0	–	44.0	12.0
Stain/solvent	11.5	13.0	48.0	50.0	20.0	48.0
Regular physical activity (% valid)						
Yes	34.6	26.1	50.0	36.4	36.0	76.0
No	65.4	73.9	50.0	63.6	60.0	24.0
Smoking (% valid)						
Yes	69.2	56.5	25.0	45.5	52.0	52.0
No	30.8	43.5	75.0	54.5	44.0	48.0
Etilism (% valid)						
Yes	30.7	21.7	50.0	22.7	36.0	40.0
No	69.2	78.3	50.0	77.3	64.0	60.0
Vegetable consumption (% valid)						
Yes	76.9	91.3	100	81.8	96.0	100
No	23.2	8.7	–	18.2	4.0	–
Meat consumption (% valid)						
Yes	92.3	100	100	95.5	100	100
No	7.7	–	–	4.5	–	–

WG (Without Gastritis, n = 32); (Without Gastritis + OME, n = 24), G (Gastritis, n = 26); G + OME (Gastritis + OME, n = 26), G + HP (Gastritis + *H. pylori*, n = 22); G + HP + OME (Gastritis + *H. pylori* + OME, n = 22)

^a^The term *pardo* refers to Brazilians of mixed ethnic ancestries

**Fig. 1:**
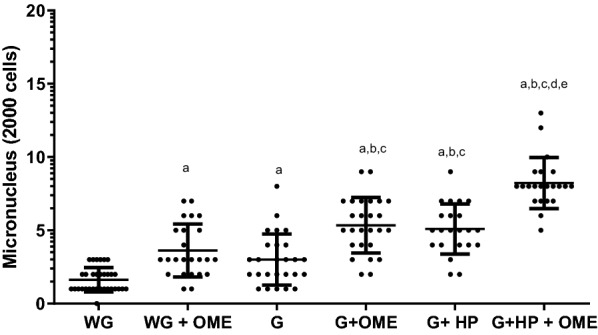
Clastogenic and/or aneugenic effects involved in micronuclei formation in 2000 stomach epithelial cells. **A** MN, **B** binucleated, WG (Without Gastritis, n = 32); (Without Gastritis + OME, n = 24), G (Gastritis-n = 26); G + OME (Gastritis + OME, n = 26), G + HP (Gastritis + *H. pylori*, n = 22); WG + OME G + HP + OME (Gastritis + *H. pylori* + OME, n = 22). Values represent the mean ± S.D.M. Differences between groups were determined by Analysis of Variance (ANOVA), and Bonferroni’s test (post hoc test). Significances were observed for < 0.05 and p < 0.0001, **a** in relation to WG, **b** in relation to SG + OME, **c** in relation to G, **d** in relation to G + OME and **e** in relation to G + HP

Regarding the mutagenic evaluation of patients stomach epithelium cells (antrum and body), clastogenic and/or aneugenic effects were observed through MN formation in WG + OME (3.62 ± 1.81) and gastritis (3, 00 ± 1.74), in relation to patients that didn’t have gastritis and were not OME user (1.62 ± 0.83). Patients with G + OME (5.091 ± 1.71) also had more MN than those with gastritis and no OME use. The presence of *H. pylori* in patients with gastritis (5.09 ± 1.71) also contributed to these effects, which in OME therapy presented increased MN formation (8.22 ± 1.74), in relation to all study groups (Fig. 1). There were no significant differences between patients with gastritis in relation to those without gastritis and in prolonged OME use, as well as among those with G + HP in relation to those with G + OME.

**Fig. 2:**
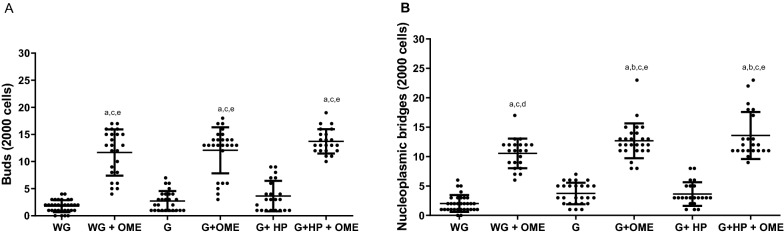
Buds (**A**) and Nucleoplasmic bridges (**B**) formation in 2000 oral epithelial cells. WG (Without Gastritis, n = 32); (Without Gastritis + OME, n = 24), G (Gastritis-n = 26); G + OME (Gastritis + OME, n = 26), G + HP (Gastritis + *H. pylori*, n = 22); WG + OME G + HP + OME (Gastritis + *H. pylori* + OME, n = 22). Values represent the mean ± S.E.M. Differences between groups were determined by Analysis of Variance (ANOVA), and Bonferroni’s test (post hoc test). Significances were observed for < 0.05 and p < 0.0001, **a**, **b**, **c**, **d**, and **e** in relation to WG, SG + OME, G, and G + OME, and **e** G + HP, respectively

OME therapy was able to induce other nuclear alterations such as buds and nucleoplasmic bridges on the stomach epithelium (body and antrum) (Fig. 2). Increased number of buds were observed in patients without gastritis (11.67 ± 4.26) and with gastritis (12.08 ± 4.26) when compared with patients without gastritis and OME use (1.78 ± 1, 09). The presence of *H. pylori* in patients with gastritis (3.69 ± 2.80) did not increase buds in comparison to those with gastritis without OME use (2.13 ± 1.80), but when in therapy with OME (13.73 ± 2.25), an increase of buds was detected, indicating effects on expression and genes (Fig. 2A).

In relation to nucleoplasmic bridges, OME induced an increase in patients without gastritis (10, 55 ± 2.52) and with gastritis (12.69 ± 2.96) when compared to the group without gastritis and OME use (2.03 ± 1.42). In patients with gastritis (3.73 ± 1.82) and gastritis and *H. pylori* (3.65 ± 2.01), it was not seen an increase in nucleoplasmic bridges in relation to the group without gastritis and no OME use. However, in patients with *H. pylori* and OME use (13.59 ± 3.99) increased nuclear abnormalities were detected (Fig. 2B).

**Fig. 3:**
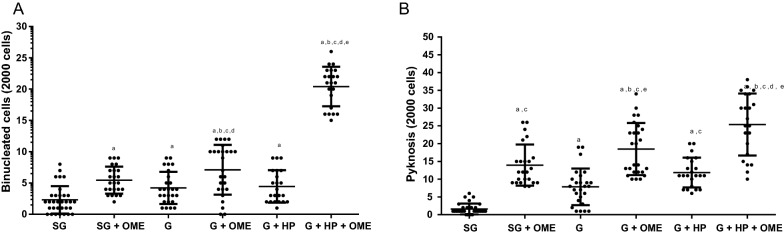
Cytogenetic changes indicative of cytotoxicity in the stomach epithelium of patients under omeprazole (OME) therapy by the formation of **A** binucleated cells and **B** pyknosis. WG (Without Gastritis, n = 32); WG + OME (Without Gastritis + OME, n = 24), G (Gastritis-n = 26); G + OME (Gastritis + OME, n = 26), G + HP (Gastritis + *H. pylori*, n = 22); G + HP + OME (Gastritis + *H. pylori* + OME, n = 22). Values represent the mean ± S.D.M. The differences between the groups were determined by Analysis of Variance (ANOVA), followed by the Bonferroni test (post hoc test). Significances were observed for p < 0.0001, **a**, **b**, **c**, **d**, and **e** in relatated to SG, SG + OME, G, **d** G + OME, and G + HP, respectively

The OME use and/or therapy induced cytotoxic effects by increasing binucleated cells in patients without gastritis and in OME user without medical prescription (5.44 ± 2.14). In addition, cytotoxicicity was also observed in patients with gastritis (7.11 ± 3.97), as well as in patients with gastritis and positive for *H. pylori* (20, 41 ± 3.15), when compared to patients without gastritis and no OME use (2.33 ± 0.91). Moreover, similar results were found in patients with gastritis, *H. pylori* and no OME use (5.01 ± 1.27). Likewise, in patients with gastritis with/without OME use (4.23 ± 1.25), an increase in binucleated cells was observed (Fig. 3A).

OME also induced cytotoxic effects by pycnotic cells formationin patients without gastritis and OME use (13.96 ± 5.17) and in patients with gastritis and OME use (18.46 ± 4.32), as well as in patients that are not using OME, but positive for *H. pylori* (7.84 ± 3.16) and in *H. pylori* positive patients and OME therapy (23, 36 ± 8.72), when compared to patients without gastritis and are not using OME (1, 61 ± 0.61) (Fig. 3B).

**Fig. 4:**
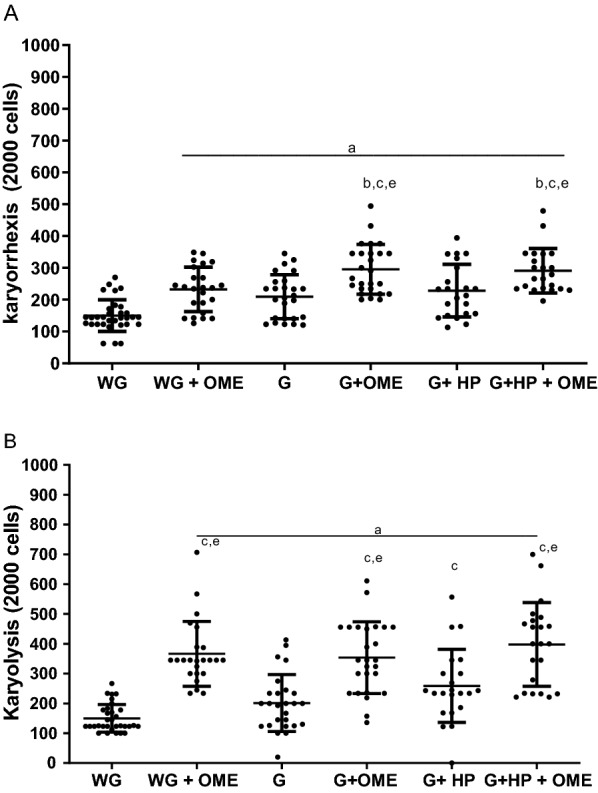
Cytogenetic changes indicative of apoptosis in the stomach epithelium of patients undergoing Omeprazole therapy (OME) by the formation of **A** karyorexis and **B** karyolysis. WG (Without Gastritis, n = 32); WG + OME (Without Gastritis + OME, n = 24), G (Gastritis-n = 26); G + OME (Gastritis + OME, n = 26), G + HP (Gastritis + *H. pylori*, n = 22); G + HP + OME (Gastritis + *H. pylori* + OME, n = 22). Values represent the mean ± S.D.M. Differences between groups were determined by Analysis of Variance (ANOVA, followed by the Bonferroni test (post hoc test). Significances for p < 0.0001. **a**–**e** in relation to WG, WG + OME, G, G + OME, and G + HP, respectively

**Fig. 5:**
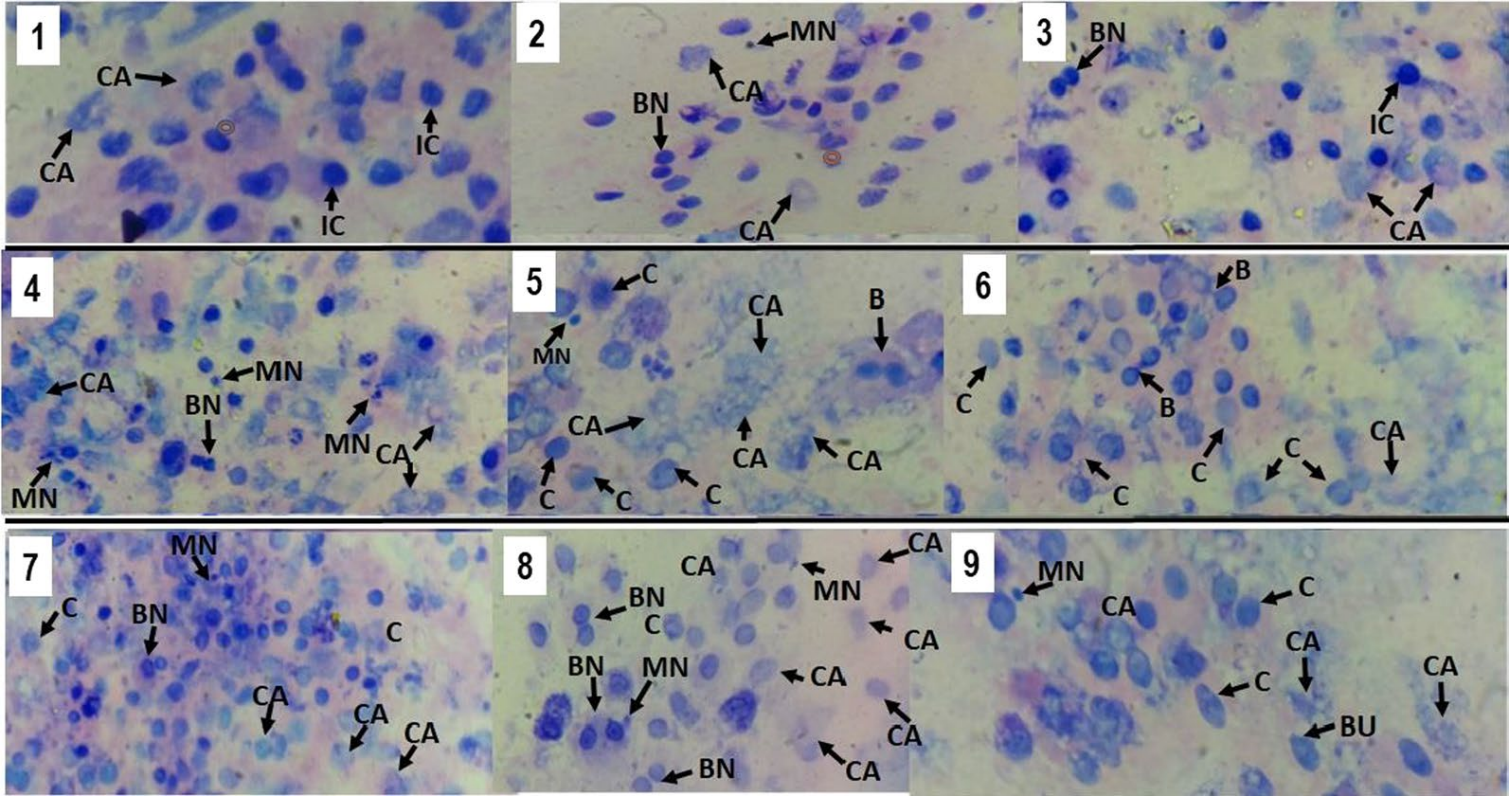
Photomicrographic profile (light microscopy-1000 × magnification, Giemsa staining) of cytogenetic analyzes of cells obtained from stomach biopsies of patients with gastritis (1–3); with gastritis treated with omeprazole (4–6); with gastritis and *H. pylori* on omeprazole treatment (7–9). In slides (1–3) intact chromatin (IC) is observed, but with some nuclear alterations. In slides (4–9) greater amounts of cell death and cytogenetic alterations are observed. Legend: Micronuclei (MN); bridges (B); karyorrhexis (C); karyolysis (CA); Buds (BU); binucleated (BN)

Similar to observed for binucleated and pycnotic cells, OME induced apoptotic effects by nuclear fragmentation (karyorrhexis) in stomach epithelium cells of patients without gastritis (202.20 ± 69.65), and with gastritis and no *H. pylori* (232, 60 ± 93.63) or with *H. pylori* (209, 40 ± 78.06), when compared to patients without gastritis and no OME use (150.00 ± 49.02). The presence of *H. pylori* in patients with gastritis and on OME therapy (291, 10 ± 70.20) also induced an increase in cariorrexis (Fig. 4A). Apoptotic OME effects by nuclear dissolution (karyolysis) were similarly observed in stomach epithelial cells of patients without gastritis (366.30 ± 108.60), with gastritis in OME use (359.00 ± 120.20) and with gastritis, *H. pylori* and OME use (397.80 ± 140.50) in relation to patients without gastritis and no OME use (149.90 ± 46.32). However, these effects were also observed in patients with gastritis (324.20 ± 179.40) and with gastritis, *H. pylori* (258.00 ± 122.70) and no OME therapy (Fig. 4B). A photomicrographic profile with nuclear and mutagenic changes can be seen in Fig. 5, emphasizing mainly the groups treated with omeprazole.

**Fig. 6:**
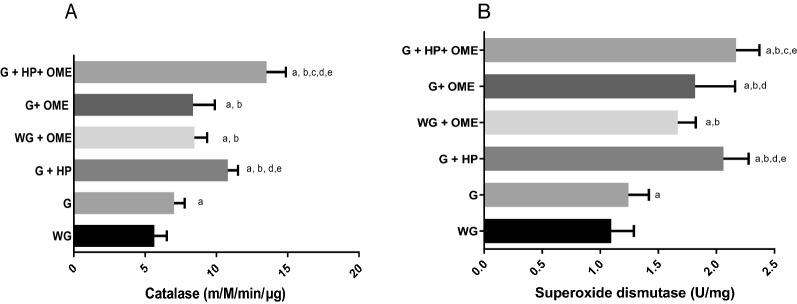
Changes in antioxidant enzyme dosages during omeprazole therapy (OME-40 mg) in patients with negative and positive gastritis. **A** Catalase and **B** superoxide dismutase. WG (Without Gastritis, n = 46); (Without Gastritis + OME, n = 22), G (Gastritis-n = 48); G + OME (Gastritis + OME, n = 48), G + HP (Gastritis + *H. pylori*, n = 27); WG + OME G + HP + OME (Gastritis + *H. pylori* + OME, n = 27). Values represent the mean ± S.D.M. Differences between groups were determined by Analysis of Variance (ANOVA, followed by the Bonferroni test (post hoc test). Significances were observed for p < 0.05 and p < 0.0001, **a**, **b**, **c**, **d**, and **e** in relation to WG, G, G + HP, WG + OME, and G + OME, respectively

In the study, it was observed that OME induces changes in dosages of antioxidant enzymes such as catalase and superoxide dismutase (Fig. 6). In patients without and with gastritis under OME therapy or were in therapy, as well as in patients with gastritis without/with*H. pylori*, they presented catalase increases in relation to patients without gastritis and were not taking OME (Fig. 6A). However, patients with gastritis and *H. pylori* on OME therapy showed significant increase in catalase compared to the other groups. The data for superoxide dismutase were similar to those observed for catalase (Fig. 6B).

**Fig. 7:**
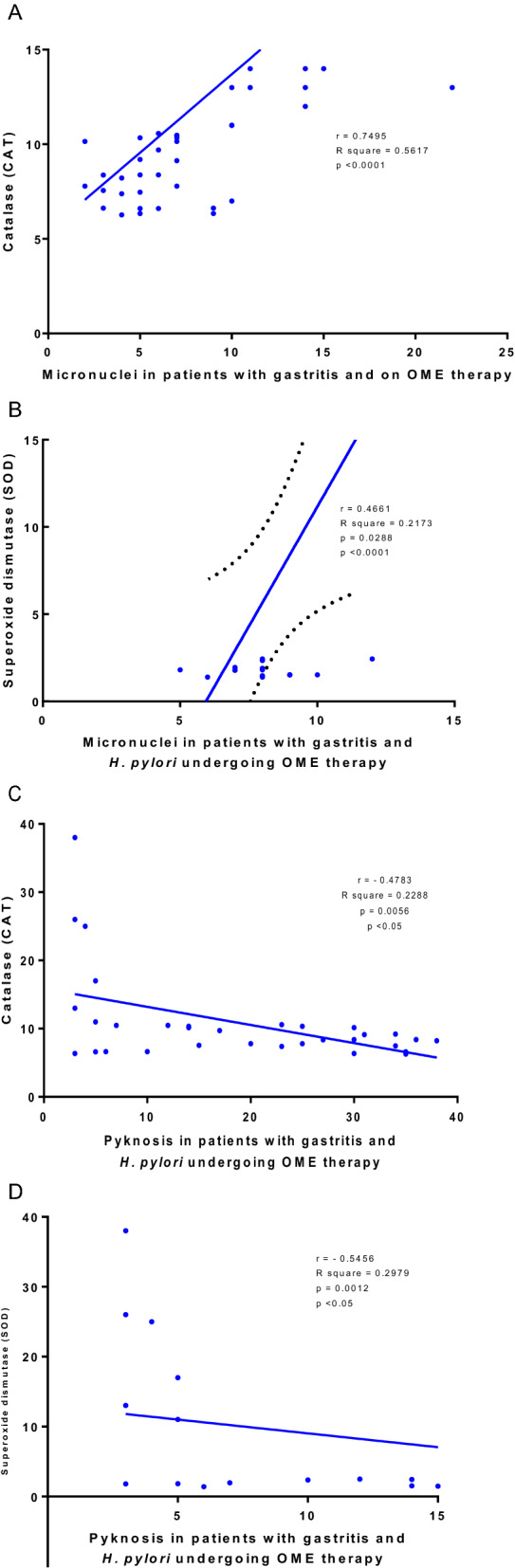
Pearson’s statistical correlations between cytogenetic biomarkers and antioxidant enzymes for micronuclei and catalase (**A**); micronuclei and superoxide dismutase (**B**); pycnosis and catalase (**C**) and **D** pycnosis and superoxide dismutases (**D**). WG (Without Gastritis, n = 46); (Without Gastritis + OME, n = 22), G (Gastritis-n = 48); G + OME (Gastritis + OME, n = 48), G + HP (Gastritis + *H. pylori*, n = 27); WG + OME G + HP + OME (Gastritis + *H.* *pylori* + OME, n = 27)

In patients with gastritis and *H. pylori* on OME therapy, positive correlations were observed between micronucleus induction with catalase and superoxide dismutase measurements, and negative correlation for picnoses induction (Fig. 7).

## Discussion

In the present study, it was observed that long term OMEuse and/or therapy, regardless dose specifications (20, 30 and 40 mg/kg) can cause cytogenetic changes in stomach epithelial cells by aneugenic and/or clastogenic effects through micronuclei formation. The OME effects on micronuclei formation in human cells are still unwell described. However, there are in silico studies suggesting that OME can lead to chromosomal changes, as well as contribute to micronuclei formation [85], also promoting DNA covalent bonds, characterizing its genotoxicity [78].

Investigations in gastric epithelial cells of patients with gastritis and with positive *H. pylori* infection, indicate a risk of genotoxicity, with greater significance in relation to negative patients [62], as well as was observed in the study in relation to the formation of micronuclei, in epithelial cells of the stomach. *H. pylori* infections can cause chronic gastritis, peptic and duodenal ulcers, adenocarcinoma and gastric lymphoma [99]. There are reports that OME can induce DNA damage, after its metabolism by N-nitrosamines formation, generating several nuclear alterations such as MN, pycnosis and cariorrexis [69, 96]. MN can also be induced by chromosome breaks [84], internal chromosomes that have been separated from the nucleus [10], double-stranded DNA breaks or as a result of mitotic spindle dysfunction [34, 35]. It is worthynote that DNA breaks-induced apoptosis can also happen due to oxidative stress [32, 79].

According to Amieva and Peek [4], there are two bacterial factors that make it a risk factor for gastric cancer and peptide ulcer: (1) the oncoprotein CagA that stimulates cell proliferation by mitotic signaling; (2) the cytotoxin VacA that alters membrane permeability and causes mitochondrial injury-induced apoptosis. In addition, other virulence mechanisms are pointed out, such as motility (flagella), chemotaxis, urease production (pH neutralization) and adaptive mechanisms such as inflammation activation, immune suppression, E-cadherin cleavage and host cell cholesterol modification [7].

According to Raza et al. [83], the *H.* *pylori* virulence is determined by the presence of the cytotoxin associated with the CagA gene, VacA cytotoxin and SabA adhesion proteins. The VacA cytotoxin induces membrane channel formation, cytochrome C release and modulates antigen presentation [39]. SabA acts as a chemical gradient (bicarbonate/CO_2_ or urea/ammonia) that guides the bacteria to bind the mucosa [76]. SabA expression is associated with intestinal metaplasia development, gastric atrophy and cancer [3, 54, 102].

DNA damage can be induced by OME through its secondary metabolites sulfone, sulfite and hydroxy-omeprazole [28, 85], as also for its electrophilic potential [82] due to covalent DNA bonds [18, 38]. It is observed that OME can increase nuclear cell proliferation antigens (PCNA) [57, 109], by modulating lysosomal transport, with mechanisms of LC3 gene expression associated to autophagy [101].

In addition to micronuclei formation, other cytogenetic cahnges were observed in the stomach epithelial cells of patients with/without gastritis (with/without *H. pylori* infection, in and/or OME therapy), such as nucleoplasmic buds and bridges. Tthe buds are the result of DNA amplification or repair [35, 60] and the bridges are originated from failures in chromosomal rearrangements or are result from chromosomal ends fusion, telomeres that allow chromatin filaments formation of that link two distinct nuclei [35]. Corroborating the findings in this study, previous reports show that OME can induce changes in chromosomes and micronucleus formation [18, 38, 85].

The OME cytotoxicity has been reported in normal human cells (HEK293 and NIH3T3) [91]. In stomach epithelial cells of patients in OM|Euse or in therapy, cytogenetic changes are indicative of cytotoxicity, due to picnoses and binucleated cells formation, especially in patients with *H. pylori* infection. Pyknosis occur due to chromatin condensation and dissolution, and binucleated cells result from cytokinesis failures at the end of cell division [86]. *H. pylori* releases cytotoxins that can induce apoptosis by alterations in cytochrome C release [39], as well as destroy cellular junctions in the gastric epithelium [3], and promote transient increased acid secretion that lead to hypochlorhydria and intestinal metaplasia [99]. These events are linked to increased grastric cancer risk [51, 63, 66, 89, 90, 105].

Moreover, OME has hepatotoxic effects as a result of apoptosis stimulated by tumor necrosis factor alpha (TNF-α), as well as by alterations of liver enzymes AST and ALT [22, 37, 98]. There is also reports of nephrotoxic effects, thrombocytopenia, acute interstitial nephritis, anaphylactic reactions and gynecomastia [22, 61].

In this study, OME induced apoptosis in stomach epithelial cells due to nuclear fragmentation (cariorrexis) and nuclear dissolution (karyolysis), as seen in patients without gastritis and with gastritis in OME use and/or therapy, and also in patients with *H. pylori* infection, as previously observed in other nuclear alterations. Although apoptosisis one of the mechanisms associated to acute gastric injury [59], OME has apoptotic effects in human gastric cancer cells (HGC-27) [108], colorectal tumor cells [52, 67], and normal human nuclear polymorphic leukocytes [20, 68, 72].

According to previous studies, drugs that induce oxidative damage may increase the levels of endogenous antioxidant enzymes such as catalase and superoxide dismutase [2, 47, 80]. Catalase (CAT) is one of the antioxidant enzymes that participates in H_2_O_2_ degradation through dismutation reactions, mainly in peroxisomes, and it has been considered as an important oxidative biomarker [45, 77]. Superoxide dismutase (SOD) converts the oxygen produced during oxidative stress to H_2_O_2_. In this regard, to act effectively in maintaining cellular integrity and function, SOD depends on the balance between SOD, GPx and CAT [74, 77].

In this study it was possible to detect an increase in antioxidant defenses for these enzymes, especially in patients with *H. pylori* infection. *H. pylori* infection can also increase reactive oxygen and nitrogen species in the stomach [42]. Moreover, gastric lesions can induce oxidative stress, with amplification by OME therapy [53] independently of co-infection with *H. pylori*, and also increase antioxidant enzymes such as SOD and CAT, and glutathione reductase (GSH) [9, 41]. Drugs may contribute to increase oxidative stress levels [2, 47, 81], due to an imbalance between antioxidant defenses and oxidative stress levels [44] and regulation of lipid peroxidation [104].

OME is one of the drugs that can induce oxidative stress [53], which culminates in cell apoptosis [77, 92, 106]. Free radicals induce gastric lesions [93], and contribute to carcinogenesis [100]. Gastric lesions can produce free radicals, which are controled by SOD and GPx enzymes, which lead to tissue recovery and gastroprotection [24, 25]. OME can also induce lipid peroxidation, and as a cellular response, increased activity of catalase and superoxide dismutase is observed, which make them, important oxidative stress markers [24].

During metabolism, OME can generate sulfone, sulfite and hydroxy-omeprazole, compounds that can generate more oxidative damage [14, 15, 28]. OME increase heme-oxigenease enzyme independently of the aryl hydrocarbon receptor (AhR), which consequently increases peroxide levels [73]. Oxidative damage can be one of OME mechanisms for inducing DNA changes in gastric epithelium cells, as it can produce H_2_O_2_ when it binds to protein C283, which contains CACT and C136 for generating beta oxidation of fatty acids [97]. OME can induce oxidative damage in *S. cerevisiae*, in addition to cytogenetic damage in murine Sarcoma 180 cells [50, 75].

**Fig. 8:**
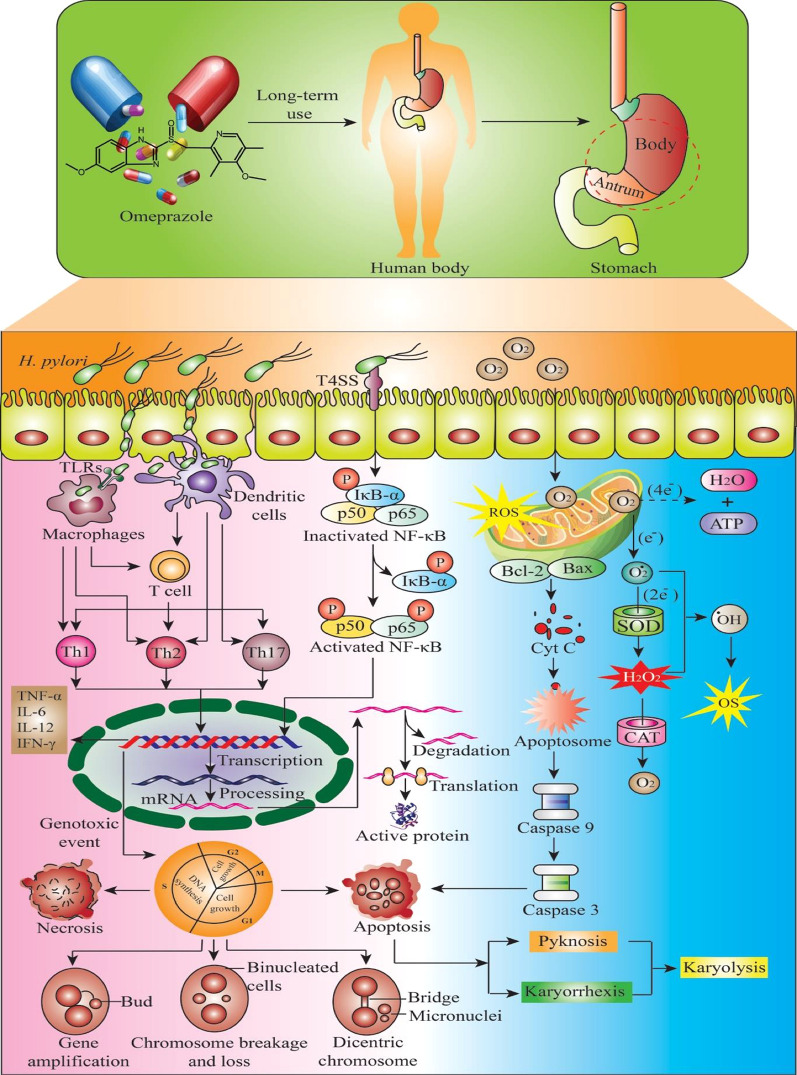
Long-term omeprazole use possible induces mutagenic, apoptoses and necroses effects through oxidative stress induction that can lead to micronuclei formation andother nuclear alterations indicative of cytotoxicity and apoptosis; several cellular mechanisms induce cell regulation and activation of signaling cascades for cell death (apoptosis or necrosis), or, conversely, induce cell proliferation, metastasis, resistance to apoptosis and angiogenesis as a consequence of genetic instability. (ATP-adenosine triphosphate; CAT-catalase; IκBα, nuclear factor of kappa light polypeptide gene enhancer in B-cells inhibitor, alpha; NF-κB-nuclear factor kappa-light-chain-enhancer of activated B cells; OS-oxidative stress; SOD-superoxide dismutase)

Among these mechanisms, our study points out that cytogenetic changes can be induced by oxidative effects that lead to micronuclei formation and other nuclear alterations indicative of cytotoxicity. Corroborating these analyzes, positive and negative statistical correlations were observed between micronuclei and CAT/SOD concentrations, and between pyknosis, respectively. Proton pump inhibiting drugs (PPIs), such as OME, may have genotoxic and/or carcinogenic effects [28] through several mechanisms including oxidative stress. When substances that induce oxidative stress are in excess and the antioxidant system is unable to neutralize the oxidative process [56], several mechanisms can induce cellular regulation and activation of cell death signaling cascades (apoptosis or necrosis) [106], or, conversely, induce cell proliferation, metastasis, resistance to apoptosis and angiogenesis as a consequence of genetic instability (Fig. 8) [12, 19, 65].

## Conclusions

In this study, in stomach epithelial cells of patients without gastritis and with gastritis, especially those with *H. pylori* infection, and OME use and/or therapy, it was possible to point out that OME induces cytogenetic changes due to (1) clastogenic and/or aneugenic effects that induce micronuclei formation; (2) altered gene expression, chromosomal rearrangements and fusion of chromosomal ends; (3) cytotoxicicity by increased picnoses and binucleated cells and (4) apoptosis by increasing karyorexis and karyolysis. Several mechanisms, not yet elucidated, can be attributed to these OME cytogenetic effects, but oxidative effects can also be involved, as observed by increased concentrations of endogenous antioxidant enzymes such as catalse and superoxide dismutase, which have also been associated to increased micronuclei and picnoses. These data point out the risks regarding long term OME use/therapy, as well as the monitoring of cytogenetic changes and oxidative damage, as an important strategy for the genetic instability prevention.

## Data Availability

Yes.
